# Smart city construction and new-type urbanization quality improvement

**DOI:** 10.1038/s41598-023-48490-x

**Published:** 2023-11-29

**Authors:** RongJun Zhou, Siqi Chen, Bingbing Zhang

**Affiliations:** 1 School of Economics and law, Chaohu University, Hefei, 238000 China; 2 Party School of Liandu District Committee of CPC, Lishui, 323000 Zhejiang China; 3 School of Economics and Management, Nanjing Agricultural University, Nanjing, 210095 Jiangsu China; 4 China Academy of Resources, Environment and Development, Nanjing Agricultural University, Nanjing, 210095 Jiangsu China

**Keywords:** Ecology, Environmental social sciences

## Abstract

First, utilising text quantitative analysis techniques, this paper analyses the smart city pilot policy in depth and clarifies its theoretical mechanism that influence the quality of new-type urbanisation. The revised entropy technique is then used to calculate the new-type urbanisation quality of 276 Chinese cities with a prefecture level or higher from 2007 to 2018. The above action mechanism is evaluated using the Difference-in-Difference model, employing the smart city pilot policy as a quasi-natural experiment (DID). The results indicate that the implementation of the smart city pilot policy can significantly enhance the quality of new-type urbanisation, and this conclusion is robust under a variety of conditions, including parallel trend testing, tendency score matching, exclusion of other policy interference and placebo testing. The analysis of heterogeneity indicates that the smart city pilot strategy has a greater impact on the qualitative improvement of new-type urbanisation in historic industrial bases, resource-based cities, and large-scale cities. The mechanism test confirmed that the construction of smart cities has improved the quality of new-type urbanisation primarily through the optimization and upgrading of industrial structure brought about by smart industrial policy and scientific and technological innovation fostered by smart government and smart people's livelihood policies.

## Introduction

Due to forty years of reform and opening, China's urbanisation level has increased rapidly. China's urbanisation rate reached 63.9% in 2020, increasing by 1.03% points annually on average. However, growing urbanisation has also placed substantial strain on regional population, resources, and ecological carrying capacity^1^. The “megacity malady” has been steadily growing, and issues like as food security, environmental pollution, climate change, and energy use have gained prominence^2^. As the rate of urbanisation increases, the question of how to effectively address the issue of sustainable urban development has become essential. In this environment, a new sort of urbanisation plan was developed. The National New-type Urbanization Plan (2014–2020) recommends integrating information technology into the entire urban development process and pursuing an intensive, intelligent, environmentally friendly, and low-carbon new-type urbanisation. The new style of urbanisation pursues the coordinated development of the economy, population, society, and environment, as opposed to the old urbanisation development model. It is an urbanisation that places greater emphasis on the quality and sustainable growth of urbanisation. Scientific measure, therefore, the new-type urbanisation quality, further to explore the key factors of the new-type urbanisation, not only has a certain academic value at the theoretical level, but also at the practical level for expanding domestic demand, fully tapping the local large market potential, building the new economic development paradigm of “dual-circulation” to realise the economic decision-making basis and reference for the development of high-quality urbanisation.

The smart city pilot policy is an effective way to cope with urban sprawl, excessive energy consumption and environmental pollution, and an important measure to improve the quality of life of urban residents. Smart city construction can increase urban operation efficiency and improve the quality of life of urban people using ICT and other digital information technologies^3^. Building a smart operation centre with “urban brain” at its core on the foundation of ICT intelligent services may change conventional cities into more intelligent and green sustainable cities, and enhance the quality of life for urban people in every way. As a result, when the Ministry of Housing and Urban Rural Development announced the list of the third batch of pilot cities, it included smart communities intimately tied to the quality of life of residents as a need for the development of smart cities. This also demonstrates that the smart city pilot strategy is highly aligned with the "people-oriented" notion of new-type urbanisation. In light of this, the issue of this article is whether the pilot policy for smart city building has achieved its key goals and enhanced the quality of new-type urbanisation. If yes, what is the exact mechanism? In view of this, this study uses the text quantitative analysis method to examine the smart city pilot policy in depth, and try to elucidate the mechanism and transmission channel of the smart city pilot policy impacting the quality of new-type urbanisation. This can not only enrich the relevant research on causal relationship identification of smart city pilot policies, but it can also provide a practical path option for “Improving the new-type urbanisation strategy and improving the quality of urbanisation development” as proposed in the “Thirteenth Five Year Plan.”

Different from previous research, this paper's contribution consists of: first, using text quantitative analysis methods such as policy document retrieval, *simhash* [simhash algorithm is a fingerprint recognition technology, which belongs to local sensitive hash. It was proposed by GoogleMoses Charikar and applied to mass text weight removal. Its main idea is dimensionality reduction. According to the experimental verification, for the 64-bit simhash value, the similarity is relatively high if the hamming distance is less than 3.] De duplication, *jieba* [jieba segmentation is a popular Chinese text segmentation tool used to segment Chinese text by word or phrase. It is an open source library written in Python that can help with Chinese text processing tasks such as natural language processing (NLP), text analysis, and information retrieval.] Word segmentation and word frequency statistics to analyse the smart city pilot policy in depth; and second, dividing the smart city pilot policy into three dimensions: smart government, smart industry, and smart people's livelihood, which provides a visual reference for scientists. Second, using panel data of 276 cities above prefecture level in China from 2007 to 2018, the improved entropy method is used to comprehensively measure and evaluate the quality of new-type urbanisation along five dimensions: population, economy, infrastructure and public services, quality of life, resources, and environment. Thirdly, from the perspective of smart city, we examined its specific impact on new-type urbanisation, clarified the theoretical mechanism for smart city construction to enhance the quality of new-type urbanisation, and provided new empirical evidence for promoting China's new infrastructure, new-type urbanisation initiatives, and major projects.

## Literature review

Current study on new-type urbanisation focuses mostly on three elements. Firstly, research on the measurement of the level of new urbanization and its driving factors. Traditional measures of urbanization level often use a single indicator, such as population urbanization rate^4,5^ Obviously, the information covered by this indicator is relatively small and cannot truly reflect the quality of urbanization. Afterwards, the measurement of urbanization level gradually shifted from a single indicator to a multi indicator evaluation method that considers many factors such as population, economy, society, environment, and space. Cai et al.^6^ constructed comprehensive evaluation indicators for urbanization from four dimensions: population urbanization, economic urbanization, social urbanization, and spatial urbanization, enriching the connotation of new urbanization. Xiong Xianghui and Xu Zhangyong combined factor analysis with principal component analysis and determined indicator weights to measure the level of urbanization from six aspects: population urbanization, economic urbanization, infrastructure equalization, public service equalization, quality of life urbanization, and resource environment^7^. Based on the correlation analysis of spatial indicators, a comprehensive indicator system was constructed to comprehensively reflect the level of urbanization. However, it should be pointed out that the prerequisite for principal component analysis is that the cumulative contribution rate of the first few principal components extracted reaches a high level and can provide explanations that are in line with the actual background and significance, otherwise the principal components are meaningless. Therefore, when extracting principal components, some information is often lost. With the continuous advancement of relevant research, entropy method, as an objective weighting method that can ensure that the information of all indicators is not missed, has gradually been adopted by many scholars. Wu^8^ used the entropy method to select 26 indicators from four aspects: economy, society, environment, and urban–rural coordination, and constructed a comprehensive indicator to measure the level of new urbanization in prefecture level cities, filling the shortcomings of previous studies. Similarly, Yu^9^ constructed a comprehensive indicator for measuring the level of new urbanization at the provincial level, following the principles of scientificity, precision, and comparability.

In terms of research on the driving factors of new urbanization, many scholars have discussed the impact of economic factors on new urbanization. The research by Xianghui and Zhangyong^10^ found that financial support is an important factor affecting the level of new urbanization. Bo and Fengchao^11^ used statistical data from 1995 to 2012 as samples to explore the relationship between technological innovation and new urbanization, and found that there is a short-term lag effect in the impact response of technological innovation on urbanization. Wu et al.^12^ used provincial panel data spatial econometrics to empirically study the impact of industrial structure adjustment on new urbanization. The study showed that both rationalization and upgrading of industrial structure can significantly promote the quality of new urbanization. In addition, studies have shown that agglomeration of productive service industries, FDI, and poverty alleviation are all important factors affecting new urbanization^8,13–15^. The academic community has also explored the relationship between government behavior and new urbanization from an institutional perspective. Xiaoyu and Liutang^16^ introduced the agglomeration effect of enterprises and land finance into the urban economic model, discussed the impact of land transfer behavior on local industrialization and urbanization, and analyzed that the optimal land transfer strategy of urban local governments is an important reason to attract enterprises, promote production, and promote urbanization. Wen et al.^17^ constructed an endogenous urbanization model that includes heterogeneous labor forces in rural and urban areas, as well as land use in rural and urban areas, studying the impact of land system and registered residence system reform on urbanization and residents' welfare, the study found that the decline in the friction between land transfer and labor migration linked to urban construction land indicators can help improve the level of urbanization. Zhihui and Hui^18^ analyzed the impact of fiscal decentralization on the development of new urbanization based on panel data from 31 provinces (autonomous regions, municipalities) from 2007 to 2018. The study found that fiscal decentralization has a significant promoting effect on the development of new urbanization. The more financial resources local governments receive, the higher the level of new urbanization development.

The second is related research on smart city construction. Currently, academic research on smart cities has focused on its conceptual framework, development path, and technology service application, with less systematic research focusing on the socio-economic effects it causes^19–21^. Jianjun and Xinjing^22^ explored the industrial structure upgrading effect of smart cities by studying human capital and incorporating it into the theoretical framework of the impact of smart city construction on industrial structure upgrading. The results showed that smart city construction will promote the rationalization and upgrading of industrial structure, which is conducive to the transformation and upgrading of industrial structure. Min et al.^23^ examined the impact of smart city construction on industrial structure from three aspects: rationalization of industrial structure, level of specialization, and level of service industry production. The study found that there are significant differences in the impact of smart city construction on industrial structure in different regions. In addition, the construction of smart cities not only has a positive impact on the upgrading of industrial structure, but also is conducive to improving the total factor productivity of enterprises and improving regional social capital^24,25^. There are also studies indicating that the construction of smart cities has a significant promoting effect on green development. Daqian et al.^26^ used the smart city pilot policy for the first time to evaluate its impact on environmental pollution, enriching literature on environmental pollution influencing factors. The results showed that the construction of smart cities has promoted innovation in urban development models using modern information technology, and reduced urban environmental pollution through innovation driven technological, configuration, and structural effects^27^. Wang et al.^28^ found that smart city oriented regional management can improve the efficiency of urban land green utilization through the development of information industry and regional innovation capabilities. Scholars have also used the multi-period double difference method to empirically explore the impact of different batches of smart city construction on the quality of urban development. Research has shown that the pilot construction of smart cities has significantly improved the quality of urban development^29^. It can be found that there has been relatively comprehensive research on the construction of smart cities in the academic community, but few studies have examined the internal mechanism of its impact on the quality of new urbanization from the perspective of smart city pilot policies.

The third method is text quantitative analysis. Quantitative analysis refers to identifying words that appear more frequently in a text, defining them as keywords or core words, and continuously mining the hidden information behind the text. Some scholars use existing documents and literature to conduct quantitative analysis of government related policies^30,31^. Huiping and Ning^32^ collected 189 policy articles from government portal websites and used policy text analysis methods to find that in the era of big data, the development logic of government governance upgrading in China can be divided into three stages: preliminary stage, top-level design stage, and comprehensive exploration stage. In the comprehensive exploration stage, the advantages of big data should be fully utilized for specific scenarios, potential should be deeply explored, and information security and property ownership should be emphasized, Create more public value. Huang and Luk^33^ constructed a monetary policy uncertainty index based on the top ten mainstream newspapers in China using text analysis. Hongyi and Qiongwen^34^, based on the observation data of manufacturing enterprises from 2013 to 2020, used text analysis and word frequency statistical techniques to measure digital innovation in enterprises, and constructed a comprehensive indicator system for green development of manufacturing enterprises from three dimensions: economic profit, social value, and environmental benefits. Hu et al.^35^ conducted a textual analysis of smart city policy documents from 341 prefecture level cities in China from 2009 to 2020, and found that cities with more developed and densely populated economies often place technology at the center of smart city decision-making, as do cities in the early and late stages of smart city development. From this, it can be seen that there are few literature that uses textual quantitative analysis to deeply analyze smart city pilot policies, and specifically divides them into three dimensions: smart government, smart industry, and smart people's livelihood, accurately identifying their differential impact paths on new urbanization.

By summarising and classifying the existing literature, it can be determined that the existing research results have been relatively rich, which also provides a rich theoretical basis and multi-dimensional analysis perspective for the relevant research in this paper; however, there are still some limitations: First, current research have thoroughly covered smart city development and new urbanisation, but few studies have explored the internal mechanism and transmission channels of the influence of smart city pilot policies on the quality of new urbanisation. Existing studies primarily focus on the smart city construction itself, such as the development path of smart cities and the application of technical services, and use the DID model to determine the impact of smart city construction on industrial structure upgrading, urban innovation, and even pollution emission. However, few studies have used quantitative analysis methods of policy texts to analyse the multiple dimensions of smart city pilot policies and to thoroughly investigate them.

## Theoretical analysis framework

A smart city is an advanced stage of urban digitalization that applies the concept of the Internet of things and supports municipal operations with little human–computer interaction^3^. Theoretically, the implementation of smart city pilot policies can fully coordinate the information sharing mechanism and specialised production factors, promote the continuous output of the digital information technology innovation system, create smart industry clusters, and enhance the quality of new urbanisation. Specifically, governments at all levels use cloud platforms, big data, dynamic sensors, and other technologies to achieve autonomous, accurate, and real-time information perception during the creation of smart cities. Alternatively, the government realises information bearing and transmission through the Internet of Things and 5G, and realises information processing and integration within the city based on the formation of various application service systems, such as urban government affairs, public security, and people's livelihood, in order to provide a highly perceptible urban basic environment for the construction of new urbanisation. Simultaneously, the synergistic effect of IT talent, professional knowledge, new information infrastructure, and other production factors that match the development of smart industry is effectively played out and gradually transformed to a higher level in order to provide factor guarantee for the new urbanisation. The formation and efficient output of intelligent innovation systems, such as innovation in digital information knowledge, technical innovation, and management system innovation, provide institutional assurance for the development of modern urbanism. In addition, relevant government departments emphasise the construction of new and efficient digital infrastructure through the construction of network infrastructure in specific regions, investigate new industrial models of digital industrialization and industrial digitalization, and form smart industry clusters in order to continuously improve the quality of new urbanisation. The following possibilities are therefore proposed:

### Hypothesis 1

The effective implementation of smart city pilot policies is conducive to improving the quality of new-type urbanization.

As a complete city-level policy covering many dimensions such as government affairs, industry, and people's livelihood, the smart city pilot policy is separated into three aspects in practise: smart government affairs, smart industry, and smart people's livelihood^36^. The smart industry policy reflects the competitive advantages given by the fourth industrial revolution. It mainly exploits the availability and accessibility of information technology to improve conventional favourable sectors and establish strategic emerging industries to achieve green production and efficient production. Specifically, on the one hand, smart agriculture can use new technologies, new materials and new energy to improve agricultural production efficiency, and use modern information systems and technologies to provide effective information support for agricultural production, supply and marketing and related management and services to enhance agricultural management efficiency. On the other side, smart agriculture may make current scientific and cultural knowledge flow into farmers through the transmission and integration of information, increase the quality of farmers, and then promote agricultural modernization. Smart manufacturing uses sensor technologies, cloud computing, deep learning, big data, etc. to connect smart machines with employees^37^, and rapidly create the Industrial Internet on the basis of achieving smart systems, smart devices, and smart decision-making. At the same time, the rise of a series of strategic emerging industries such as nanomaterials, equipment manufacturing, and environmental protection materials, as well as the application of clean technologies such as low consumption and low emissions, can optimise and upgrade traditional advantageous industries and promote accelerated industrial transformation and upgrading. In addition, vigorously build information infrastructure to develop basic services; rely on information network technology to develop smart logistics, smart finance, e-commerce and other production and market services, nurture smart tourism, smart entertainment and other personal smart consumer services, thereby promoting the modernization of the service industry, intelligent, and then promote the optimization and upgrading of the industrial structure. The green production and high-efficiency production brought about by the optimization and upgrading of the industrial structure would not only minimise environmental pollution, but also promote high-quality economic development. This is consistent with the core connotation of new-type urbanization, that is, the improvement of the quality of new-type urbanization pays attention to the efficiency of resource utilization, environmental friendliness and ecological balance. By encouraging the development of green technologies and sustainable industries, smart industry policies promote the evolution of urban industrial structure to a more environmentally friendly and sustainable direction, and ultimately help to improve the quality of new-type urbanization. Accordingly, the following hypothesis is put forward:

### Hypothesis 2

The smart industry policy in the pilot implementation of smart cities can improve the quality of new-type urbanization by promoting the optimization and upgrading of the industrial structure.

Smart government policies adopted in smart city projects are related to the efficiency, transparency of government administrative structures, and public participation in decision-making^38^, a field that emphasises successful cooperation and interaction in big metropolitan networks. The smart governance of the government encourages the public to strengthen the supervision and participation of government information and behaviour, reduce the information asymmetry between the government and enterprises, the government and the public, and the public and enterprises, and compel enterprises to engage in technological innovation. The implementation of e-government will facilitate the strengthening of environmental regulations in smart cities^36^, facilitate the connection between citizens and local governments in order to better meet the needs of residents^39^, and improve the transparency and accountability of public services^40,41^. When people believe that environmental pollution is intensifying and impacting their quality of life, they will offer feedback via the online service platform, and the online monitoring platform will oversee the execution. Under the pressure of public oversight and participation, the government will compel businesses to make corrections within a specified period of time. In addition, in response to pressure from both the government and the public, businesses typically boost their R&D expenditures in order to reduce pollution emissions. The wise people's livelihood policy seeks to enhance the public's standard of life by providing comprehensive public infrastructure and high-quality public services. In the science and technology innovation chain paradigm, regardless of whether it is original technology or disruptive technology, basic research is the primary source of technological advancement. Nonetheless, the allocation structure among funds, talent, environment, and innovation aspects is crucial to the formation of the fundamental research mechanism's dynamic nature. The execution of the smart people's livelihood policy ensures a good standard of living for scientific researchers, and numerous intelligent platforms and service systems have improved the comfort and convenience of the scientific research environment. This form of financial and environmental assistance enables eminent scientists to devote themselves to fundamental research, encourage fundamental scientific and technical innovation. In summary, smart government and smart livelihood policies, through technological innovation, have optimized government efficiency, improved public services, enhanced residents' quality of life, and fostered urban sustainability, thereby incorporating the essence and features of new-type urbanization quality. These policy mechanisms contribute to the creation of smarter, more livable, and sustainable cities, boosting urban competitiveness and residents' quality of life, ultimately enhancing the quality of new-type urbanization. Consequently, the following theory is proposed:

### Hypothesis 3

Smart government policy and smart livelihood policy in the pilot implementation of smart cities can improve the quality of new-type urbanization through technological innovation.

**Figure 1:**
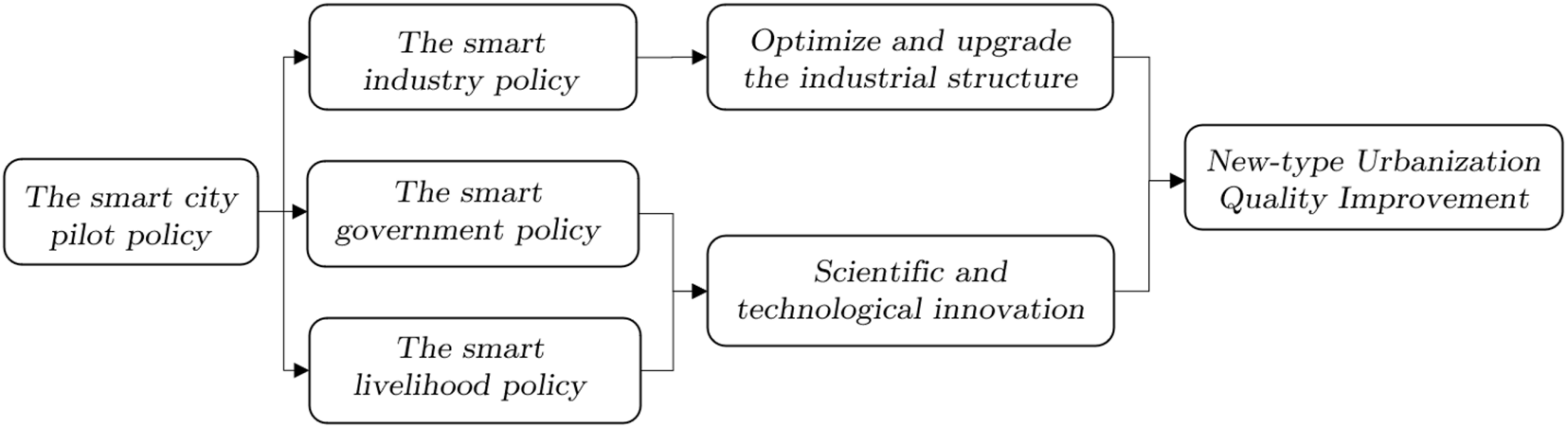
Operation mechanism.

Figure 1 shows the mechanism of the impact of smart city pilot policies on the quality of new-type urbanization.

## Model construction and variable selection

### Construction of the econometric model

This article will use the multi-period DID model approach to determine the effect of the smart city pilot policy on the quality of new-type urbanisation, based on a theoretical analysis of the policy.

1 $$new\_urb_{it} = \beta_{0} + \beta_{1} treat_{i} \times period_{t} + \rho X_{it} + \delta_{i} + \mu_{t} + \varepsilon_{it}$$

where *new_urb*_*it*_ represents the new-type urbanization quality of city *i* in year *t*; *treat*_*i*_ is a grouping dummy variable, *period*_*t*_ is a period dummy variable, and the interaction term *treat*_*i*_ × *period*_*t*_ is the net effect of policy implementation; *X*_*it*_ is a set of control variables, including regional openness, income level, medical and health infrastructure, technological progress and employment structure; *δ*_*i*_ is the city fixed effect, *μ*_*t*_ is the time fixed effect represents the random disturbance term.

### Variable selection and calculation

#### Dependent Variable: quality of new-type urbanization

This paper employs the enhanced entropy method to evaluate the quality of urbanisation of the new kind. The entropy approach is a form of objective weighting. The classic entropy method is limited to processing cross-sectional data and assigning unique weights to each tiny index. The new style of urbanisation is human-centered, seeks the integrated growth of economy, people, society, and environment, and places an emphasis on urbanization's quality and sustainability. This paper improves the traditional entropy method by incorporating time variables to make it suitable for panel data. It then determines the weight of each indicator and calculates the comprehensive score, thereby constructing a structure that includes population structure, economic development, infrastructure, and public services. A complete measurement index system for the quality of new-type urbanisation comprised of five categories and eighteen subcategories, such as service, quality of life, and resources and environment. The comprehensive measurement index system of new-type urbanization quality is shown in Table 1.

**Table 1:** Comprehensive measurement index system of new-type urbanization quality.

Indicator categories	Indicator subcategory	Representative variable	Unit
Population structure	*pop_den*	Population density	%
	*stu*	The number of students in general undergraduate colleges	Per person
	*unemp*	The number of registered unemployed persons in cities and towns	Per person
Economic development	*pgdp*	GDP per capita	Ten thousand yuan
	*invest*	Fixed asset investment	Ten thousand yuan
	*ter_gdp*	The tertiary industry's share of GDP	%
Infrastructure and public services	*pub_bus*	There are buses and trams per 10,000 people	Unit
	*road*	Paved road area per capita	m^2^
	*green*	Green coverage in built-up areas	%
	*end_ins*	The number of people participating in the basic endowment insurance for urban workers	Per person
	*medicare*	The number of people insured by the basic medical insurance for urban workers	Per person
Quality of life	*pbook*	Public library collections per capita	Volume
	*network*	Number of Internet Broadband Access Users	10,000 households
	*mobile*	Number of mobile phone users at the end of the year	10,000 households
Resources and environment	*ref_dis*	Harmless treatment rate of domestic waste	%
	*sew_dis*	Centralized treatment rate of sewage treatment plant	%
	*SO*_*2*_	Industrial SO2 Emissions	t
	*N*_*x*_*O*_*y*_	Industrial NOx Emissions	t

#### Core explanatory variable: smart city pilot policies

This paper's primary explanatory variable is the smart city pilot policy (*treat* × *period*). Among them, *treat* is a dummy variable that indicates whether or not the policy is a smart city pilot programme. If the city is a pilot city, *treat* = 1 is assigned to it; otherwise, *treat* = 0 is assigned; period is before and after the policy pilot. The *period* dummy variable is set to 0 prior to the policy implementation year and 1 during and after that year. Multiplying the pilot group dummy variable by the time dummy variable yields the interaction term *treat* × *period*, which is the principal explanatory variable for the smart city pilot policy's net effect.

#### Selection of control variables

The control variables include: ① The actual amount of foreign direct investment attracted by prefecture-level cities, as expressed by the logarithm of the urban openness index (*lnfdi*). Generally speaking, the bigger the quantity of foreign direct investment a city draws, the more open the city is. ② Income level (*lnpay*), which is determined by the logarithm of the average wage of urban workers. The amount of income is a significant factor influencing new-type urbanisation. ③ Medical and health infrastructure (*lnnum*), as assessed by the number of hospital beds and health centre beds in urban areas. The perfection of a city's medical infrastructure frequently influences the quality of life and life satisfaction of urban residents and is a significant element influencing the quality of urbanisation. ④ Technological advancement (*lntech*) is measured by the logarithm of the number of inventive patents awarded by metropolitan businesses. The greater a city's innovation vitality, which is also helpful to enhancing the quality of urbanisation, the more patents it has. ⑤ Employment structure (*nemp_prop*), the employment structure of an area is determined by the percentage of workers in the tertiary industry. A suitable and ideal employment structure facilitates the growth of advanced industries, which in turn influences the quality of new-type urbanisation.

The data used in this study are sourced from the “China Urban Statistical Yearbook,” and “China Environmental Statistical Yearbook” spanning from 2007 to 2018. The “China Urban Statistical Yearbook” provides comprehensive information about urban development in various regions of China, including data related to urban population, employment, economy, society, environment, and other aspects. The “China Environmental Statistical Yearbook” offers more detailed basic data concerning environmental conditions in different provinces, autonomous regions, and municipalities across China. This data encompasses environmental quality, pollutant emissions, environmental protection, and environmental management, among other factors. The data for constructing the various indicators of new-type urbanization quality and the control variables required for regression analysis were all obtained from the the “China Urban Statistical Yearbook,” and “China Environmental Statistical Yearbook.” Due to significant data gaps in the statistical yearbooks for the Tibet Autonomous Region and certain cities, we excluded cities with substantial missing data. As a result, we collected relevant data from 276 prefecture-level cities in China for the years 2007 to 2018, which were then organized into a balanced panel dataset to ensure data integrity and consistency. The descriptive statistics of main variables are shown in Table 2.

**Table 2:** Descriptive statistics of main variables.

Value		Obs	Mean	St. dev.	Min.	Max.
Dependent variable	The quality of new-type urbanization (*new_urb*)	3312	0.139	0.0887	0.0347	0.867
Control variables	The actual amount of foreign investment (*lnfdi*)	3312	9.809	1.858	1.099	14.55
	number of beds in hospital (*lnnum*)	3312	9.487	0.683	7.355	11.82
	Proportion of employees in the tertiary industry (*nemp_prop*)	3312	0.528	0.131	0.0991	0.948
	Number of invention patents granted (*lntech*)	3312	3.750	0.794	0.293	4.615
	Average salary of employees (*lnpay*)	3312	10.58	0.431	8.509	12.68

## Analysis of empirical results

### Benchmark regression results

The results of the benchmark regression are reported in Table 3. Column (1) represents the estimation result when just the core explanatory factors and fixed effects are included, but not the control variables. The calculated coefficient of the pilot policy dummy variable may be found to be significantly positive. Table 3 columns (2–6) display the results of the benchmark regression with the incremental addition of control variables, while holding the city fixed effect and the year fixed effect constant. It is evident that the estimated coefficient of the pilot policy dummy variable is 0.004, remaining statistically significant at the 10% confidence level. This implies that the smart city pilot policy has led to an enhancement of 0.004 in the quality of new-type urbanization, approximately a 3% improvement on the mean level.

**Table 3:** The benchmark regression.

Value	*new_urb*					
	(1)	(2)	(3)	(4)	(5)	(6)
*Treat* × *period*	0.0040*	0.0040*	0.0043**	0.0041*	0.0039*	0.0040*
	(1.83)	(1.83)	(1.98)	(1.83)	(1.76)	(1.79)
*nemp_prop*		0.0173*	0.0197**	0.0201**	0.0207**	0.0199**
		(1.77)	(2.06)	(2.12)	(2.19)	(2.10)
*lnnum*			0.0118***	0.0111***	0.0109***	0.0103***
			(3.00)	(3.03)	(2.98)	(2.79)
*lnfdi*				0.0008	0.0008	0.0008
				(1.05)	(1.03)	(1.07)
*lntech*					0.0015**	0.0015**
					(2.31)	(2.34)
*lnpay*						0.0086*
						(1.73)
*_cons*	0.1691***	0.1599***	0.0515	0.0497	0.0459	-0.0330
	(113.44)	(28.95)	(1.51)	(1.40)	(1.30)	(− 0.59)
Sample size	3312	3312	3312	3312	3312	3312
City fixed effects	Y	Y	Y	Y	Y	Y
Year fixed effects	Y	Y	Y	Y	Y	Y
R^2^	0.390	0.391	0.394	0.396	0.396	0.397

***, **, and *represent significance at the 1%, 5%, and 10% levels, respectively, with t values in parentheses, and robust standard errors clustered to the city level.

Using the column (6) regression findings as a baseline, examine the control variables. The computed *nemp_prop* coefficients are significantly positive. Therefore, the increase in the share of workers in the tertiary sector will improve the employment structure and the quality of new-type urbanisation. The coefficients are positive and pass the significance test at the 1% level, according to *lnnum*. This demonstrates that the more comprehensive the medical and health infrastructure, the better the life and health of residents can be ensured, which is favourable to developing people's livelihood and enhancing the quality of new-type urbanisation. At the 5% significance level, the predicted coefficient for *lntech* is considerably positive. This indicates that cities with higher technology levels have a greater number of new company formats, which are more favourable to enhancing the quality of urbanisation of new types. The computed lnpay coefficient is positive and statistically significant. This demonstrates that an increase in worker wages is also beneficial to enhancing the quality of new-type urbanisation.

### Robustness test

#### Parallel trend test

**Figure 2:**
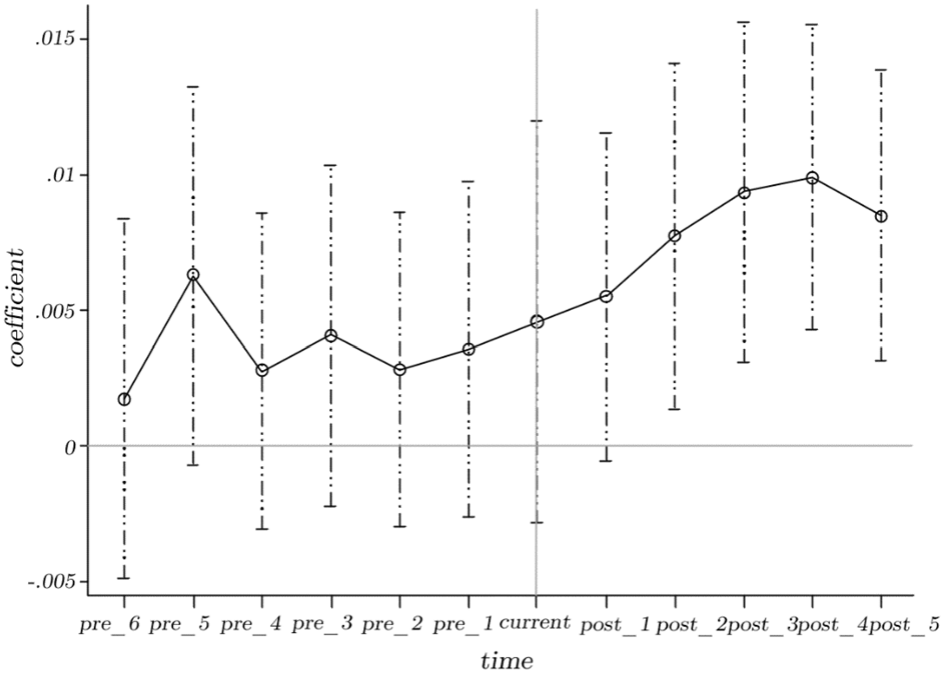
Parallel trend test.

In order to employ the difference-in-difference model, the experimental group and the control group must satisfy the assumption of parallel trends. This paper used the event study approach to examine the parallel trend, and the results are presented in Fig. 2. It can be observed that there is no substantial difference between pilot cities and non-pilot cities prior to the adoption of the programme, thereby confirming the parallel trend assumption. One year after the adoption of the policy, the quality of new-type urbanisation has demonstrated a considerable improvement trend, indicating that the influence of smart city pilot policies on the quality of new-type urbanisation is notably positive, with a one-year lag.

#### PSM–DID

In order to reduce the possibility of sample selection bias, the PSM–DID [The PSM–DID method uses the dummy variable treat, which indicates whether it is a smart city pilot policy, to perform logit regression on the observable variables, including income level, medical and health infrastructure, technology level, and employment structure, in order to obtain the matching propensity score. To reduce systematic disparities in the quality of new urbanisation among cities, the cities with the closest matching values of propensity scores were designated as the matching cities of smart city pilot programmes, i.e. the control group.] Method was employed to conduct robustness tests in this study. Specifically, in this work, the nearest neighbour matching approach is utilised for matching, and the DID method is used to examine the impact of smart city pilot policies on the quality of new-type urbanisation based on the successfully matched samples. The estimation results of the PSM–DID approach are displayed in Table 4, columns (1) and (2). Only the fundamental explanatory variables are represented in column (1). The computed coefficients of the pilot policy dummy variables are statistically significantly positive. The regression result after incorporating the control variable is shown in column (2). The estimated coefficient of the pilot policy dummy variable is found to be significantly positive. This demonstrates that the smart city pilot policy is conducive to enhancing the quality of urbanisation of a new type, and the conclusion is robust.

**Table 4:** Robustness test 1.

Value	*new_urb*				
	PSM–DID		Exclude other policy interference		
	(1)	(2)	(3)	(4)	(5)
*treat* × *period*	0.0044*	0.0041*	0.0040*	0.0041*	0.0039*
	(1.83)	(1.70)	(1.76)	(1.85)	(1.76)
*lowc*			− 0.0000		
			(− 0.00)		
*creat*				− 0.0037	
				(− 1.05)	
*sponge*					0.0034
					(0.56)
*_cons*	0.1725***	− 0.0238	− 0.0330	− 0.0293	− 0.0307
	(92.52)	(− 0.37)	(− 0.59)	(− 0.53)	(− 0.55)
Sample size	2,328	2328	3,312	3,312	3,312
Control variables	F	Y	Y	Y	Y
City fixed effects	Y	Y	Y	Y	Y
Year fixed effects	Y	Y	Y	Y	Y
R^2^	0.373	0.381	0.397	0.398	0.398

***, **, and *represent significance at the 1%, 5%, and 10% levels, respectively, with t values in parentheses, and robust standard errors clustered to the city level. The control variables are identical to those in Table 3. The subsequent tables are identical.

#### Exclude other policy interference

The net policy effect of smart city pilots may also be impacted by other policies, particularly urban management, energy conservation, and emission reduction programmes closely tied to new-type urbanisation. To eliminate interference from such policies, this part controls the Low Carbon City Pilot (*lowc*), the National Innovative City Pilot (*creat*) and the Sponge City (*sponge*) and other policies. The estimation outcomes after eliminating the aforementioned policy interference are presented in columns (3) through (5) of Table 4. The calculated coefficient of the pilot policy dummy variable is still highly positive, as can be shown. This demonstrates that low-carbon city pilots, innovative city pilots, and sponge city pilots have not altered the positive effect of smart city pilot policies on the quality of new-type urbanisation.

#### Placebo test

The prerequisite for employing the double difference method is that the experimental group and the control group must exhibit comparability, meaning that in the absence of the smart city pilot policy, there would be no significant differences in the quality of new-type urbanization over time between the experimental group and the control group. Therefore, to examine whether the improvement in new-type urbanization quality indeed stems from the smart city pilot policy, this section establishes a counterfactual policy occurrence time, advances the implementation time of the smart city pilot policy, multiplies it by the group dummy variable, and reintroduces the interaction term into the baseline regression model. If the coefficient of the counterfactual policy interaction term is significant, it indicates that even in the absence of the smart city pilot policy, other factors have enhanced the quality of new-type urbanization. If the coefficient of the counterfactual policy interaction term is not significant, it demonstrates that the smart city pilot policy has indeed had a positive effect, thus verifying the robustness of the baseline regression results. Observing the items (1–4) of Table 5, it can be seen that the estimated coefficients of the pilot policy dummy variables are not significant when the policy implementation period is advanced by one year, two years, three years, or four years. This suggests that the smart city pilot strategy has no discernible effect on the quality of new-type urbanisation in the experimental and control groups. Moreover, it implies that the actual year of policy implementation can definitely increase the quality of new-type urbanisation and that the conclusion is robust.

**Table 5:** Robustness test 2.

Value	*new_urb*			
	(1)	(2)	(3)	(4)
*treat* × *period*	0.0032	0.0026	0.0026	0.0018
	(1.56)	(1.27)	(1.27)	(0.83)
*_cons*	− 0.0309	− 0.0292	− 0.0276	− 0.0271
	(− 0.55)	(− 0.52)	(− 0.50)	(− 0.48)
Sample size	3312	3312	3312	3312
Control variables	Y	Y	Y	Y
City fixed effects	Y	Y	Y	Y
Year fixed effects	Y	Y	Y	Y
R^2^	0.397	0.396	0.396	0.396

### Heterogeneity analysis

The “National Old Industrial Base Adjustment and Reconstruction Plan (2013–2022)” splits a total of 95 cities with an old industrial base. To explore the varied influence of smart pilot city policies on the quality of new-type urbanisation, this paper separates the sample cities into old industrial base cities and non-old industrial base cities. The results of the regression are displayed in columns (1) and (2) of Table 6. It can be observed that, compared to non-old industrial base cities, the estimated coefficient of the pilot policy dummy variable for old industrial base cities is 0.0072 and is significantly positive at the 5% level. This is equivalent to an average increase of 5.4% in the quality of new-type urbanization, indicating that the smart city pilot policy is effective in enhancing the quality of new-type urbanization in old industrial base cities. In general, the antiquated infrastructure, trailing service industry level, and unsuitable urban functions of the old industrial base cities contribute to their low urbanisation quality. After smart city building is empowered, the improvement effect is more significant.

**Table 6:** Heterogeneity analysis.

Value	*new_urb*					
	(1)	(2)	(3)	(4)	(5)	(6)
	old industrial base cities	non-old industrial base cities	resource-based cities	non-resource-based cities	large-scale cities	small-scale cities
*treat* × *period*	0.0072**	0.0023	0.0064**	0.0027	0.0051*	0.0035
	(2.10)	(0.88)	(2.06)	(0.93)	(1.69)	(1.11)
*_cons*	− 0.1439*	0.0203	− 0.2098***	0.0640	− 0.0536	− 0.0222
	(− 1.73)	(0.35)	(− 3.20)	(1.03)	(− 0.61)	(− 0.35)
Sample size	1104	2208	1344	1968	1812	1500
Control variables	Y	Y	Y	Y	Y	Y
City fixed effects	Y	Y	Y	Y	Y	Y
Year fixed effects	Y	Y	Y	Y	Y	Y
R^2^	0.460	0.397	0.424	0.423	0.448	0.384

The resource endowment of a city will influence the resource utilisation efficiency and industrial structure of economic entities, which will in turn influence the city's sustainable growth. In this regard, this paper classifies all sample cities as resource-based cities or non-resource-based cities based on the notification document of the National Sustainable Development Plan for Resource-Based Cities (2013–2020) and investigates the heterogeneity of the impact of smart city pilot policies. Observing the column (3) and (4) of Table 6, it can be seen that the estimated coefficient of the pilot policy dummy variable for resource-based cities is 0.0064 and is significantly positive at the 5% level. This is equivalent to an average increase of 4.6% in the quality of new-type urbanization. However, the estimated coefficient for non-resource-based cities is not significant. This indicates that the smart city pilot policy has a greater impact on enhancing the quality of urbanisation of a new kind in resource-based cities. The heavy industry dominates the industrial structure of resource-based cities, and the extractive sector accounts for more than 20% of the secondary industry. Modern industrial and high-tech sectors are at a relatively low level, and reliance on natural resources is still significant. Nevertheless, after the transformation of digital information technology, relying on technological innovation to continuously develop clean energy and clean technology, promote the optimization and upgrading of the industrial structure, and then significantly enhance the quality of its new-type urbanisation.

The urban scale expansion's agglomeration impact will lead innovation elements to agglomerate, which is beneficial to the improvement of urban innovation capabilities and urban functions. Then, would the influence of smart city pilot programmes on the quality of urbanisation in cities of varying sizes be heterogeneous? In accordance with the State Council's “Notice on Adjusting the Criteria for the Classification of Cities,” this paper combines large cities, large cities of type I, large cities of type II, megacities, and megacities into large-scale cities, while the remaining cities are classified as small-scale cities. The estimated findings of the heterogeneity analysis are displayed in columns 5 and 6 of Table 6. It can be observed that the estimated coefficient of the pilot policy dummy variable for large-scale cities is 0.0051 and is significantly positive at the 10% level. This corresponds to an average increase of 3.7% in the quality of new-type urbanization. However, small-scale cities do not exhibit a significant impact. This demonstrates that the pilot policy has a major impact on the quality enhancement of urbanisation of the new type in large cities. Large cities have a strong information infrastructure, and they can exert an agglomeration effect to increase the level of scientific and technical innovation. In terms of the application of information technology in major cities, this makes the pilot construction of smart cities more directional and executable. In addition, the enhancement of innovation capacity influences the level of information technology. This type of interaction between virtuous circles makes the pilot strategy a “cherry on top” for enhancing the quality of new-type urbanisation in large-scale cities.

### Transmission mechanism test

The Smart Industry Index (*ind*), Smart Government Index (*gov*) and Smart Livelihood Index (*liv*) will be introduced in this part. First, this paper uses text analysis to objectively quantify the types of biased policies in the implementation of smart city pilot policies. The specific steps are as follows: (1) A total of 917 documents related to smart city pilot policies publicly disclosed on the websites of municipal people's governments since the implementation of the pilot policies are manually collected to form a text analysis dataset [The dataset mainly includes the following four types of documents: First, direct directional documents for the construction of smart cities, such as smart city development planning, implementation plans, construction plans, guidance opinions, notices, decisions and project management methods, etc. Second, policies with keywords as the theme closely related to smart cities, such as “big data”, “Internet of Things” “cloud computing”, “digital city”, “informatization” and so on. Third, special projects and subdivision policies for the construction of smart cities, such as “integration of the two”, “optical network city”, “information consumption”, “e-commerce”, “5G”, etc. Fourth, chapters related to the construction of smart cities in the National Economic and Social Informatization Development Plan.] And use python software to perform simhash algorithm to remove the re-load and jieba segmentation of the source files in the data set to eliminate potential noise sources. (2) Word frequency of each theme in the statistical data set quantifies the popularity of core themes in smart city construction, and divides them into three policy types: smart industry, smart government, and smart people's livelihood. Smart industry seeks to utilise the availability and accessibility of information technology to improve the quality and upgrade traditional industries, cultivate and develop strategic emerging industries to achieve green and efficient production, such as smart agriculture, smart manufacturing, modern service industry, e-commerce, artificial intelligence, big data, the Internet of things, and cloud computing. Smart government focuses on cooperation and interaction in the large network between different groups in the city, and the combination of various management agencies can serve residents more reliably and efficiently, including e-government, smart transportation, smart ecology, smart public security, and smart urban management. Smart livelihood policy includes smart medical care, smart education, smart community, smart elderly care, and smart social security in an effort to increase the contentment and happiness of its residents. (3) The entropy method is used to calculate the comprehensive indicators of the Smart Industry Index (*ind*), Smart Government Index (*gov*) and Smart people's Livelihood Index (*liv*). In addition, this part takes the value of the output value of the tertiary industry and the value of the regional innovation and entrepreneurship index as proxy variables of industrial structure upgrading and scientific and technological innovation respectively, and adopts the stepwise method to conduct mechanism test. Among them, the output value of the tertiary industry comes from the "China Urban Statistical Yearbook" over the years, and the regional innovation and entrepreneurship index comes from the Enterprise Big Data Research Center of Peking University.

The computed coefficients of smart industry policy variables are significantly positive in column (1) of Table 7. This demonstrates that the smart industry policy plays a key role in supporting the industrial structure upgrade. Also highly positive is the estimated coefficient of the industrial structure variable in column (2). This demonstrates that the modernization of industrial structure contributes to the enhancement of the quality of new-type urbanisation. Clearly, the smart industry policy implemented by the smart city pilot may promote the modernization of the industrial structure and enhance the quality of new-type urbanisation. This not only benefits the economic prosperity of individual cities but also contributes to the sustainable development of nations and regions. In columns (3) and (4) of Table 7, the estimated coefficients for the variables clever government policy and smart people's livelihood policy are positive and consistent. This indicates that smart government policies and smart people's livelihood policies have a substantial positive impact on scientific and technological innovation, which is conducive to boosting the level of urban scientific and technological innovation. In column (5), the estimated coefficient of the technical innovation variable is positive and passed the significance test at the 1% level, indicating that technological innovation can enhance the quality of new-type urbanisation. This finding underscores the pivotal role of the government in improving the quality of new-type urbanization. By promoting technological innovation and digital services, the government can deliver more efficient public services, meet the needs of citizens, and accelerate urban development.

**Table 7:** Transmission mechanism test.

Value	Mechanism I		Mechanism II		
	(1)	(2)	(3)	(4)	(5)
	Structure	new_urb	inndex	inndex	new_urb
sco_ind × period	0.0136***				
	(3.32)				
sco_gov × period			0.0147**		
			(2.45)		
sco_liv × period				0.0187***	
				(3.47)	
structure		0.0212***			
		(4.19)			
inndex					0.0028***
					(3.33)
_cons	9.0956***	− 0.2743***	− 3.7478***	0.2321	− 0.0295
	(13.73)	(− 3.38)	(− 3.83)	(0.20)	(− 0.53)
Sample size	3013	3013	3289	3289	3289
Control variables	Y	Y	Y	Y	Y
City fixed effects	Y	Y	Y	Y	Y
Year fixed effects	Y	Y	Y	Y	Y
R^2^	0.952	0.417	0.087	0.090	0.399

## Conclusions and implications

This paper firstly analyzes the mechanism of smart city pilot policies affecting the quality of new urbanization from the theoretical level; then, the improved entropy method is used to measure the quality of new-type urbanisation in China's prefecture-level and above cities from 2007 to 2018, and the smart city pilot policy is used as a natural experiment, using the difference-in-difference method The empirical test investigates the internal mechanism and transmission channel of smart city pilot policies on new-type urbanisation quality. The outcome of the study indicates that the development of smart cities contributes to the enhancement of the quality of new-type urbanization, and this conclusion has passed the robustness test in multiple scenarios. The smart city pilot strategy has a greater impact on the quality improvement of new-type urbanisation in old industrial base cities, resource-based cities, and large-scale cities, as determined by heterogeneity analysis, effect is not considerable. The mechanism test demonstrates that the smart city pilot policy promotes the optimization and upgrading of the industrial structure through the implementation of smart industry policies, smart government affairs policies, and smart people's livelihood policies in order to promote technological innovation and enhance the quality of new-type urbanisation.

This paper's contribution consists of: first, using text quantitative analysis methods such as policy document retrieval, simhash de duplication, jieba word segmentation and word frequency statistics to analyse the smart city pilot policy in depth; and second, dividing the smart city pilot policy into three dimensions: smart government, smart industry, and smart people's livelihood, which provides a visual reference for scientists. Second, using panel data of 276 cities above prefecture level in China from 2007 to 2018, the improved entropy method is used to comprehensively measure and evaluate the quality of new-type urbanisation along five dimensions: population, economy, infrastructure and public services, quality of life, resources, and environment. Thirdly, from the perspective of smart city, we examined its specific impact on new-type urbanisation, clarified the theoretical mechanism for smart city construction to enhance the quality of new-type urbanisation, and provided new empirical evidence for promoting China's new infrastructure, new-type urbanisation initiatives, and major projects.

This paper's research findings have significant policy implications: First, the multidimensional enhancement of the smart pilot policy system will continue to facilitate the overall improvement of the quality of urbanisation of the new type. For example, innovate the government's "decentralization service" model to strengthen smart governance, promote the deep integration of the digital economy and the real economy to form smart industrial clusters, build online public services and monitoring platforms to build smart livelihood systems. Second, create a smart policy framework based on local characteristics and apply precise policies to enhance the quality of urbanisation of the new type. For old industrial base cities, it is necessary to rely on digital technology to foster and support the development of clean industries, expand modern service industries, and reduce energy consumption. For resource-based cities, relying on intelligent manufacturing to crack the innovation bottleneck of all links of the industrial chain and improve the level of deep processing of resources, and build an intensive and efficient resource development pattern. Third, consistently improve the quality of new-type urbanisation by using the benefits of smart city pilot programmes and scientific and technical innovation as a robust starting point. First, continue to develop a smart and convenient environment for scientific research, maximise the scientific research strengths of universities and research institutes, bolster fundamental research, and establish a solid basis for the quality enhancement of new-type urbanisation. The second objective is to rely on digital information technology to enable the optimization and upgrading of old industries, to develop emerging digital industries, and to give new industrial support for the enhancement of the quality of new-type urbanisation. Third, with the aid of digital technologies such as the Internet and cloud platforms, new links and activities are derived in existing fields, implemented and expanded in an orderly manner, thereby forming new business formats and providing a continuous impetus for the enhancement of the quality of new-type urbanisation. Focus on market demand, break the original vertically distributed industrial chain and value chain, enabling the efficient combination of traditional industrial parts to establish a new model, and make clear path options for the qualitative improvement of new-type urbanisation.

## Data Availability

All data generated or analysed during this study are included in this published article and its supplementary information files.
